# Set-up and validation of mycobacterial interspersed repetitive unit-variable number of tandem repeat (MIRU-VNTR) analysis of *Mycobacterium tuberculosis* using BioNumerics software

**DOI:** 10.1371/journal.pone.0205336

**Published:** 2018-10-31

**Authors:** Mathias Klok Pedersen, Aase Bengaard Andersen, Dorte Bek Folkvardsen, Erik Michael Rasmussen, Erik Svensson, Troels Lillebaek, Philip Supply

**Affiliations:** 1 International Reference Laboratory of Mycobacteriology, Statens Serum Institut, Copenhagen, Denmark; 2 Department of Infectious Diseases, Copenhagen University Hospital, Copenhagen, Denmark; 3 Univ. Lille, CNRS, Inserm, CHU Lille, Institut Pasteur de Lille, U1019—UMR 8204 - CIIL - Centre d'Infection et d'Immunité de Lille, Lille, France; 4 Genoscreen, Campus de l'Institut Pasteur de Lille, Lille, France; Institut de Pharmacologie et de Biologie Structurale, FRANCE

## Abstract

The objective was to describe and validate a new and alternative software procedure for 24-locus mycobacterial interspersed repetitive unit-variable number-tandem repeat (MIRU-VNTR) typing of *Mycobacterium tuberculosis* (Mtb) based on the multipurpose BioNumerics software. DNA from randomly selected isolates of Mtb from two European laboratories, including external control samples for MIRU-VNTR typing, were analysed. Samples were genotyped using the commercial 24-locus VNTR typing kit from GenoScreen. The PCR amplified fragments were separated by capillary electrophoresis. For the subsequent analyses, the currently used software GeneMapper was compared with BioNumerics. The endpoint was the level of concordance when comparing genotyping results obtained from BioNumerics with results obtained from GeneMapper and the ECDC proficiency study reference results. Also, the number of necessary manual standard size corrections and allele assignments in the two different software methods were compared. In total, 272 DNA samples, including the ECDC proficiency panel, were analysed. For all samples, there were 100% concordance of results. For a randomly selected set of 96 samples the numbers of manual corrections needed for size standards were 199 with GeneMapper versus zero for BioNumerics. The numbers of manual corrections for allele assignments were 122 with GeneMapper versus 16 with BioNumerics. In conclusion, we have validated the multipurpose software BioNumerics for standard 24-locus MIRU-VNTR typing and the software shows promising benefits in terms of simplification and minimization of hand-on time.

## Introduction

Tuberculosis (TB) remains a global public health hazard with estimated 10.4 million new cases and 1.4 million deaths in 2015 [1]. Despite overall declining incidences in Europe, the continent experiences 300,000–400,000 new TB cases yearly and faces challenges with multi-drug resistant strains of *Mycobacterium tuberculosis* (Mtb), which are prevalent in Eastern Europe and Asia in particular [2,3]. With political instability in European border regions and ongoing migration, control of Mtb transmission remains paramount. One key element to understand Mtb transmission dynamics and guide TB control is the genotyping of Mtb strains. Despite the development of higher resolution–but not yet standardized–methods based on whole genome sequencing, 24-locus mycobacterial interspersed repetitive unit-variable number-tandem repeat (MIRU-VNTR) typing remains widely used for genotyping because its standardization enables concerted national and international molecular surveillance of TB [3,4]. In multiple TB reference laboratories globally, MIRU-VNTR typing is performed using high throughput versions with multiplex PCR and fragment analysis on capillary sequencers [5,6]. Results from the fragment separation are analysed in the GeneMapper software, where electropherograms are visualized and processed for semi-automated allele calling [7]. However, analysing in GeneMapper is limited by the amount of manual corrections frequently needed for relabeling the size standard fragments and the subsequent allelic assignments of the MIRU-VNTR amplicons. In this study, we describe and validate a new, alternative procedure based on the multipurpose BioNumerics software [8,9] which addresses these limitations. This procedure allows integrating downstream operations completely and analysing the obtained genotypes using one single system, including databasing, attribution of genotype names synchronized with the MIRU-VNTR*plus* nomenclature [10], cluster analysis, and phylogenetic identification.

## Material and methods

Randomly selected Mtb isolates from Institut Pasteur de Lille, France and International Reference Laboratory, Statens Serum Institut, Denmark, were used for analysis. Also, as an external control, DNA samples from European Center for Disease Control (ECDC) proficiency study for MIRU-VNTR typing were used for analysis. Samples were genotyped using the commercial 24-locus VNTR typing kit from GenoScreen [11] in accordance with the manufacturer's instructions. The PCR amplified fragments were separated by capillary electrophoresis on ABI 3730 XL machines (Applied Biosystems) to determine the fragment sizes. The electrophoreses were calibrated beforehand using the calibration kit with the allelic ladder, also from GenoScreen [11]. GeneMapper version 5, Applied Biosystems, and BioNumerics version 7.6, Applied Maths, were used for the analysis in accordance with the user manuals [7,8]. The analyses of samples in GeneMapper and in BioNumerics were done independently and blindly in Copenhagen and Lille, respectively, i.e. during the analysis, there was no access to results or data from the other software. The primary endpoint was the level of concordance between results obtained from BioNumerics and results obtained from GeneMapper and the ECDC proficiency study results. The secondary endpoints were the number of manual standard size corrections and modifications of allele assignments needed in BioNumerics and GeneMapper, respectively.

The study was based on randomly selected Mtb isolates and data were accessed and analysed anonymously. As genotyping are performed as a part of national surveillance of Mtb transmission, the authors had access to identifying information in Denmark only. The study was approved by the Danish Data Protection Agency no.: 2012-54-0100. According to Danish legislation, further ethical approval and informed written consent are not required for retrospective register studies without any contact to patients.

## Results

Both the setup and the analysis using BioNumerics are described in detail in the supplemental material. In principle, BioNumerics works similarly to GeneMapper, that is i) electrophoresis data is imported as .fsa-files, ii) sizes of the size standard are specified automatically and any errors corrected manually, iii) alleles are assigned according to calculated sizes of the amplified fragments, and iv) 24-locus genotypes are obtained from aggregated alleles. The allele assignment is based on predefined sized bins, which are specified during initial setup of the database based on the experimental calibration of the sizes for different alleles of each of the 24 MIRU-VNTR loci upon electrophoresis, using the calibration kit with the allelic ladders. The attribution of existing and new genotype names is automatically done using the MIRU-VNTR plug-in, which synchronizes genotype names with the standard nomenclature and already registered genotypes maintained on the MIRU-VNTR*plus* website [12]. This is another significant difference compared to the use of GeneMapper, where the obtained genotypes have to be uploaded manually to the website for naming after genotypic analysis.

Importantly, when electrophoresis data are imported, BioNumerics appears to perform very well in automatically assigning sizes of the size standard fragments, and also perform well in the following automatic assignments of the alleles for the MIRU-VNTR amplicons and the naming of resulting genotypes, as seen from our evaluation as follows: In total 272 samples–corresponding to 1632 quadruplexes–were analysed using both GeneMapper and BioNumerics—including a set of 20 external samples from an ECDC proficiency testing round. For all samples, results obtained with BioNumerics and those obtained with GeneMapper were 100% concordant.

For a randomly selected set of 96 samples–corresponding to 576 quadruplexes–we registered the number of size standard corrections and allele assignment modifications that had to be performed manually with either software. For these samples, the number of manual corrections needed for size standards were 199 with GeneMapper versus zero for BioNumerics. The number of manual corrections for allele assignments were 122 with GeneMapper versus 16 with BioNumerics.

The total sample set (i.e. from Institut Pasteur de Lille, the International Reference Laboratory, Statens Serum Institut, and the ECDC panel) included diverse isolates from major lineages and sublineages such as EAI, CAS, Ghana, Harleem, S, LAM, Uganda, Cameroon, Beijing, H37rv, with a broad overall distribution of alleles/repeat unit numbers across the 24 loci, ranging from 0 to 16 in total.

## Discussion

In this study performed in two European laboratories, we report the evaluation and validation of the use of BioNumerics for standard 24-locus MIRU-VNTR genotyping of Mtb. We found 100% concordance with the classical use of GeneMapper, and a substantial simplification and minimization of hands-on time in the overall workflow, due to a reduction of the amount of manual modifications of both size standards and allele assignments needed. We provide specific and detailed instructions on how to setup the software and how to perform the analysis using BioNumerics as supplemental material. Our study supplements the manufacturer’s manuals, enabling implementation of a simpler and faster workflow in many laboratories currently employing high throughput MIRU-VNTR typing using GeneMapper.

It is appealing to use BioNumerics not only for the more streamlined primary analysis, but also for the fact that the subsequent data handling can be done in the same software. Thus, data can be processed by creating phylogenetic trees, maps etc., but also by connecting genotyping data to metadata such as clinical, epidemiological and whole genome sequencing data, if available. This final level of analysis is key for the epidemiological understanding of TB transmission, e.g. for delineating an outbreak or the extent of ongoing transmission in a population [13].

The analysis in BioNumerics could potentially be improved further. The main reasons for the remaining automatic misassignments of alleles were, when artefact peaks were taller than the true peak and thus falsely assigned by the software. Most frequent artefacts are “pull-up peaks” (or “bleed-through bands”), reflecting interference from other, strongly amplified fragment labeled in another colour or “stutter peaks”, caused by slippage of the DNA polymerase during PCR (**S1 File. Setup of MIRU-VNTR analysis in BioNumerics**). A setting in the software may be enabled to automatically detect “pull-up peaks”—however, this function did not appear to work satisfactorily in our study. Also, for larger fragments, the peaks were sometimes positioned just outside the predefined bin causing the peak to miss an assignment. However, this may be handled by manually increasing the size of the defined bins, when initially setting up the software.

The study has some limitations. We did not measure the actual time spent on the analysis in each software. However, the number of necessary manual corrections was much smaller when using BioNumerics than in GeneMapper. Nevertheless, the benefits of simplification and minimization of hands-on time were clear. Also, a cost-benefit analysis was beyond the scope of this study.

In conclusion, we have validated the use of the multipurpose software BioNumerics and have shown its advantage for standard 24-locus MIRU-VNTR typing.

## Supporting information

S1 File Setup of MIRU-VNTR analysis in BioNumerics. (DOCX)

S2 File Analysis of MIRU-VNTR in BioNumerics. (DOCX)
