# Supplementary material for: Set-up and validation of mycobacterial interspersed repetitive unit-variable number of tandem repeat (MIRU-VNTR) analysis of Mycobacterium tuberculosis using BioNumerics software
Source: PLoS One. 2018 Oct 31;13(10):e0205336. doi: 10.1371/journal.pone.0205336 (PMC6209162; doi:10.1371/journal.pone.0205336)
Supplement: S1 File — (DOCX) [file pone.0205336.s001.docx]

**Supplemental material I: The setup of MIRU-VNTR database in BioNumerics® version 7.6**

**Intro**

This manual describes the initial setup of the database in BioNumerics®, so it may be used to perform MIRU-VNTR analysis. The procedure itself is described in *Supplemental material II*: *The MIRU-VNTR analysis in BioNumerics® version 7.6*. The manuals are intended for laboratories using the MIRU-VNTR method. The advantage of using BioNumerics® to perform MIRU-VNTR analysis is, that the data is analysed directly in multifunctional software, where it can be further processed and used for disease surveillance and research. Also, the analysis can be made relatively easy and fast after the initially setup has been performed. The procedure may of course be refined and changed according to local laboratory settings and standing operating procedures. The manual may be regarded as a supplement to BioNumerics® own manuals and is developed by the International Reference Laboratory of Mycobacteriology at Statens Serum Institut, Copenhagen, Denmark and the Centre for Infection and Immunity, Institut Pasteur de Lille, France in collaboration.

**Contents**

1. Create database
2. Installer MIRU-VNTR plugin
3. Set bins
4. Naming of the .fsa files
5. First import of data and setup of import template
6. Back to bins to couple with quadruplexes
7. Set colors
8. Set normalization / standard sizes

**Create database**

In this step, the database used for MIRU VNTR analysis in BioNumerics® is created.

1. Open BioNumerics®.
2. Choose ”Create new database”.
3. Type a database name and click “Next”.
4. Choose “Create new”, “Next” and “Use default (SQLite)” click “Finish” (tables are created).

**Install MIRU-VNTR plugin**

Next, the MIRU-VNTR plugin must be added.

1. Choose “MIRU-VNTR” plugin and click “Activate”.
2. Upon message “Do you want to install the ‘MIRU_VNTR’ plugin” - click “Yes”.
3. When the software states “The ‘MLVA’ database components schema needs to be updated. The software will now install the current version of the database schema.

WARNING: This requires administrator privileges on the connected database. Do you want to continue?”, then click “Yes”.

1. When the software states “Provide information fields to store the MLVA types”, then check all three boxes and have the setting “Create new” for all three selections (MtbC 15-9, MtbC 15, MtbC 9). Click “OK”.


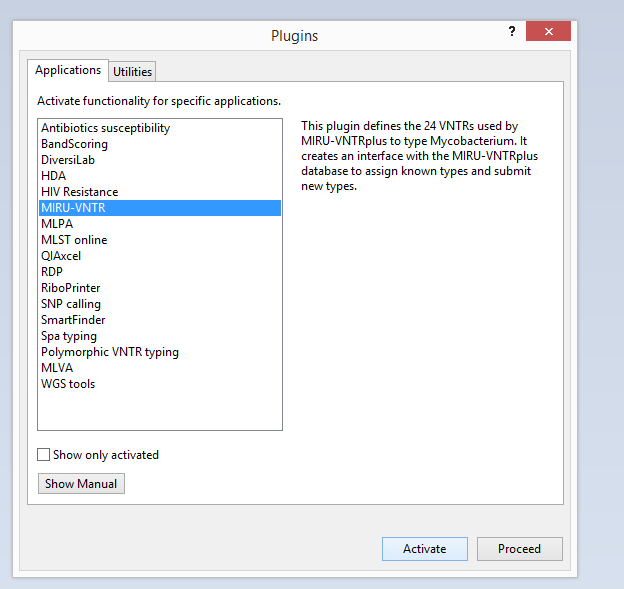


Reprinted from BioNumerics®, Applied Maths NV under a CC BY license, with permission from BioNumerics®, Applied Maths NV, original copyright 2016.

1. When the software states “Create typing field”, then let the name unchanged (MIRU_MtbC) and click “OK”.
2. When the software states “Create typing field”, then let the name unchanged (MIRU_MtbC15) and click “OK”.
3. When the software states “Create typing field”, then let the name unchanged (MIRU_MtbC9) and click “OK”.
4. The software now states that the plugin has been installed. Click “OK” and afterwards “Proceed”. You are now in the main window of BioNumerics® and your newly created database.

**Set bins**

In this step, the bin sizes will be defined. The data from the MIRU-VNTR calibration kit and validation samples run (allelic ladder) in the electrophoresis define how the bins should be set, and when performing the MIRU VNTR analysis later, the bins will define if an allele is assigned. The start of each bin is set as 10 base pairs (bp) below the validation fragment value and the stop of the bin is set as 10 bp above the validation fragment value. Some fragment sizes may be missing from the calibration data and these values have to be estimated. Use [a spreadsheet and extrapolate the series up to 15 – see example here (link)](https://docs.google.com/spreadsheets/d/1bka8smnsCcYd9Axg8BBEY6vA0AWhyJg9DnzN8rkPH7c/edit?usp=sharing). The allelic ladder for each quadruplex should increase linearly.

1. In the main window, go to “MLVA” in the menu bar and choose “MLVA management window...”
2. Choose “Edit” and then “Mappings” and then “Add mapping...”
3. If you already have the validation fragment sizes from the calibration kit as a .txt file you can import them here (alternatively as a .xml file; then you need to “Cancel” here and choose “File” and then “Import”). Choose a mapping name, e.g “Calibrated” and click “OK” and then type in the start of the bin (10 bp below the fragment value) and the stop of the bin (10 bp above the fragment value).


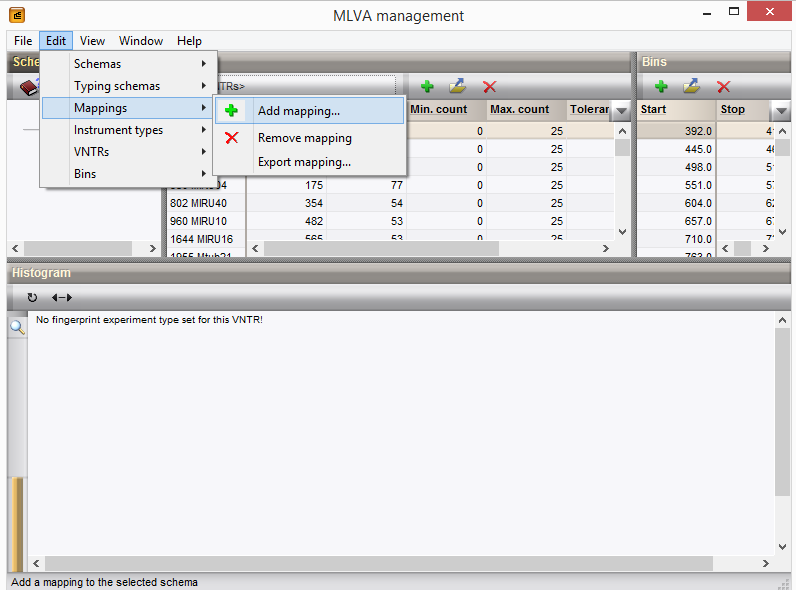


Reprinted from BioNumerics®, Applied Maths NV under a CC BY license, with permission from BioNumerics®, Applied Maths NV, original copyright 2016.

**Naming of the .fsa files**

The .fsa files are the raw data from the electrophoresis. It is important to have a routine setup, where .fsa files are named, so they can be imported correctly into BioNumerics® (otherwise you will have to change the file names manually before each import). The name you want for the “entry” and the “pools” must be separated by an underscore, for instance:

20170421johndoe_QUAD1

20170421johndoe_QUAD2

20170421johndoe_QUAD3

20170421johndoe_QUAD4

20170421johndoe_QUAD5

20170421johndoe_QUAD6

20170421janedoe_QUAD1

20170421janedoe_QUAD2

20170421janedoe_QUAD3

20170421janedoe_QUAD4

20170421janedoe_QUAD5

20170421janedoe_QUAD6

…etc.

**First import of data and setup of import template**

We need to import the first set of data before:

1. we can couple our loci and bins to a quadruplex under “MLVA” and “MLVA management window…”
2. the normalization and standard sizes can be setup, so they will work for subsequent imports as well.

So:

1. From the main window choose “File” from the menu bar and then “Import”.
2. Choose “Fingerprint type data” and then “Import curves”.
3. Click “Import” then “Browse...” and find your relevant .fsa files and mark all (“Ctrl A”). Click “Open”.
4. Provide a “Fingerprint file name” - it is best to begin with a date format of the run in this way YYYYMMDD as it is easier to sort and find data if you need them later on. Click “Next”.
5. Choose “Example import with pools” and then “Copy...”. Name the template e.g. “MIRU VNTR IMPORT” and click “OK” (keep the checkmark in “Save template in database”).
6. In next screen put a checkmark in “Show advanced options”.
   - Mark the row with destination type “Fingerprint dye” and choose “Edit parsing...” and thereafter “Preview...” The output should be 6-FAM automatically.
   - Mark the row with destination type “Fingerprint pool” and choose “Edit parsing...” and then “Preview...”. The output should be your quadruplexes - in our example “QUAD1”. If not you can manipulate the output of the data using _* (to leave things out from the filename).
   - Mark the row with destination type “Entry information” and choose “Edit parsing...” and then “Preview...”. The output should be your keys as you want them to figurate in the main window. Again: if not you can manipulate the output of the data using _*.


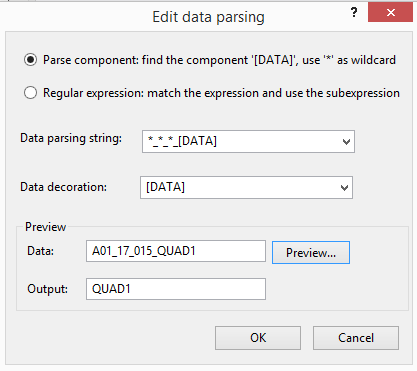


Reprinted from BioNumerics®, Applied Maths NV under a CC BY license, with permission from BioNumerics®, Applied Maths NV, original copyright 2016.

1. Click “Next” and then in next window let settings be (“Reference dye” should be LIZ and all “Dyes” should have a checkmark). Click “Next” again. In next window leave settings unchanged (there should be a checkmark next to “Key) and click “Finish”.
2. You have now defined an import template to use for subsequent imports in the database. Click next (the “Base fingerprint type” should just be kept at “<Create new>” at this stage).
3. Provide a name for the new fingerprint type, e.g. “MIRU VNTR 24”. Click “OK”.
4.
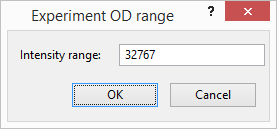
Click “Yes” to confirm.

Reprinted from BioNumerics®, Applied Maths NV under a CC BY license, with permission from BioNumerics®, Applied Maths NV, original copyright 2016.

1. Click “OK”.
2. After parsing for a moment BioNumerics® states that 30 new experiment types will be created (if is a different number, then something is wrong and you should NOT continue, but instead go back and see if you did everything correctly). Click “Yes” to continue and “Yes” again to confirm.
3. In next screen it is shown how many entries you have. This should (obviously) match with what you already know you have analysed. Click “Next”.
4. Click “Finish” and keep the checkmark next to “Open curve preprocessing window”. Now the data will be imported and a “Fingerprint curve processing Window” will open. This is where data will be further processed, but for now we need to go back to the main window in the taskbar.

**Back to bins to couple with quadruplexes**

Note that you can now see your entries in the large section to the left and the fingerprint files containing your raw data and experiment types for each quadruplex to the right. Now we need to couple the created experiment types with the relevant quadruplex. Therefore:

1. Go back to “MLVA” and “MLVA management window” in the menu bar. Choose the “Calibrated” bins to the left (below “Schemas”).
2. Choose the first locus 154 and double click.
3. Assign relevant fingerprint type and click “OK”. In this case it is MIRU VNTR 24QUAD46-FAM, corresponding to quadruplex 4 and the blue dye. This is given from the Genoscreen kit and there is no internal logic in it, but needs to be set right. You can see [which quadruplexes and dyes each locus correspond to here (link)](https://docs.google.com/spreadsheets/d/1E-NFqtfMx1_B8IUWK35J5D4wfeCMSOw0OXdhCacme3k/edit?usp=sharing).
4. Proceed with the rest of the 24 loci and close the window.


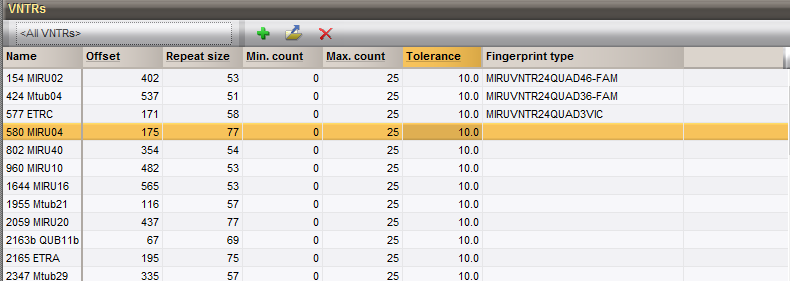


Reprinted from BioNumerics®, Applied Maths NV under a CC BY license, with permission from BioNumerics®, Applied Maths NV, original copyright 2016.

**Set colors**

In the window “Fingerprint Curve Processing”:

1. “View” in menu bar and then “Display settings”
2. For LIZ click change and choose an orange color; click “OK”
3. Repeat with other colors (6-FAM: blue, VIC: green, NED: black, PET: red)
4. Remove the checkmark in “Show single channel in black”. Click “OK”.

**
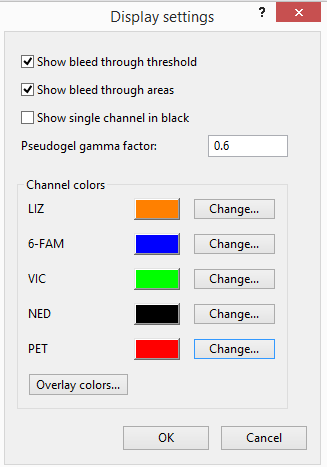
**

Reprinted from BioNumerics®, Applied Maths NV under a CC BY license, with permission from BioNumerics®, Applied Maths NV, original copyright 2016.

**Set normalization / standard sizes**

The standard sized defined will be the GeneScan 1200 LIZ® containing the fragments sizes:

80, 100, 114, 120, 140, 160, 180, 200, 214, 220, 240, 250, 260, 280, 300, 314, 320, 340, 360, 380, 400, 414, 420, 440, 460, 480, 500, 514, 520, 540, 560, 580, 600, 614, 620, 640, 660, 680, 700, 714, 720, 740, 760, 780, 800, 820, 840, 850, 860, 880, 900, 920, 940, 960, 980, 1000, 1020, 1040, 1060, 1080, 1100, 1120, 1160, 1200.

The principle is to define the standard sizes from one lane and thereafter apply it to the rest of the samples (and thereafter it will be applied to every subsequent samples imported in the database). In the window “Fingerprint Curve Processing” at the bottom left you see "Channels", where you can choose to display only certain colours of the samples, for instance when you want to analyse your standard sizes only:

1. Deselect all colors, but the orange in “Channels” (use eye icons)
2. Go to “Bands” in the menu bar and choose “Search reference bands”
3. Change “Peak detection” “% of curve range” to 1 and “% of OD range” to 0.1 - this should make sure that no peaks (bands) in the lanes are missed.
4. Click “OK”
5. Choose a lane, where the standard sizes are of good quality. From left you will have to start from fragment 80 and end with fragment 1200 to the very right in the lane. Depending of the quality of the run it means you will have to delete unnecessary peaks  (bands) often to the very left and maybe other places in the lane. You delete a peak by selecting the black triangle and type “Delete” on the keyboard. When completed, you should only have peaks in a pattern matching the GeneScan 1200 LIZ®.


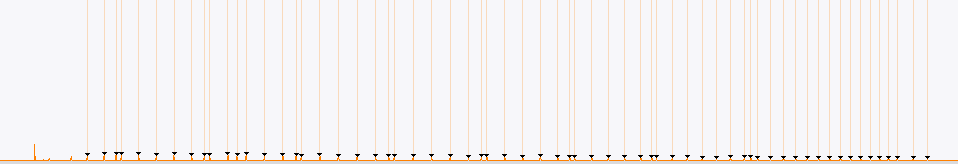


Reprinted from BioNumerics®, Applied Maths NV under a CC BY license, with permission from BioNumerics®, Applied Maths NV, original copyright 2016.

1. Choose “References” in the menu bar and the “Define size standard...”.
2. Choose GeneScan 1200 LIZ® and delete “20,30,40,60,” in the standard fragment list to the right (by simply highlighting and deleting them). Click “OK”.


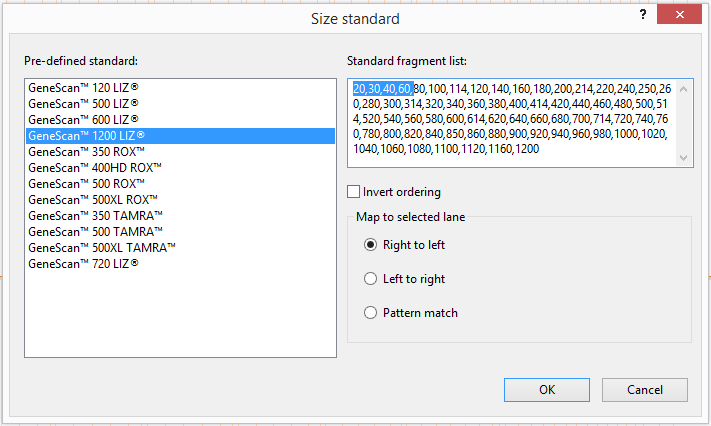


Reprinted from BioNumerics®, Applied Maths NV under a CC BY license, with permission from BioNumerics®, Applied Maths NV, original copyright 2016.

1. Choose “Normalization” in the menu bar and the “Auto assign reference positions (current)...” and leave settings default. Now, the fragment should be labeled correctly – but do spot checks.
2. Proceed to  “Normalization” in the menu bar and the “Auto assign reference positions (all lanes)...” and leave settings default.
3. Choose “File” in the menu bar and then “Save”. Now the size standard are defined for this database (both the samples you just imported and subsequent imported samples) and you have finished the database setup.  You can now analyse your data – see *Supplemental material II*: *The MIRU-VNTR analysis in BioNumerics® version 7.6*.
