# Supplementary material for: Set-up and validation of mycobacterial interspersed repetitive unit-variable number of tandem repeat (MIRU-VNTR) analysis of Mycobacterium tuberculosis using BioNumerics software
Source: PLoS One. 2018 Oct 31;13(10):e0205336. doi: 10.1371/journal.pone.0205336 (PMC6209162; doi:10.1371/journal.pone.0205336)
Supplement: S2 File — (DOCX) [file pone.0205336.s002.docx]

**Supplemental material II: The MIRU-VNTR analysis in BioNumerics® version 7.6**

**Intro**

This manual describes a procedure for using the BioNumerics® software to perform MIRU-VNTR analysis. The initial setup of the database is described in *Supplemental material I: The setup of MIRU-VNTR database in BioNumerics® version 7.6*. The manuals are intended for laboratories using the MIRU-VNTR method. The advantage of using BioNumerics® to perform MIRU-VNTR analysis is, that the data is analysed directly in multifunctional software, where it can be further processed and used for disease surveillance and research. Also, the analysis can be made relatively easy and fast after the initially setup has been performed. The procedure may of course be refined and changed according to local laboratory settings and standing operating procedures. The manual may be regarded as a supplement to BioNumerics® own manuals and is developed by the International Reference Laboratory of Mycobacteriology at Statens Serum Institut, Copenhagen, Denmark and the Centre for Infection and Immunity, Institut Pasteur de Lille, France in collaboration.

**Table of contents**

1. Start-up and import of raw data
2. Opening of previously imported data
3. Specifying standard sizes
4. Analysis of samples
5. Naming of genotypes
6. Exporting data
7. Appendix I - Artefacts
8. Appendix II - Double alleles

**Start-up and import of new raw data**

1. Open BioNumerics® via desktop icon.
2. Double-click on the relevant database.
3. Click on "File" and then "Import."
4. Select "Fingerprint type data" and then "Import curves". Click "Import".
5. Find the desired raw data via "Browse" and mark all analyses (press "Ctrl + A" and "Open").
6. Write the date in the "Fingerprint filename" in the format: YYYYMMDD (obs. this is important if you want the files to be readily searchable).
7. Click "Next".
8. Select “MIRUVNTR IMPORT” template (see manual for setup) and base fingerprint type "MIRUVNTR-24" and click "Next". Leave settings untouched in the next step and allow data to load (it may take a few minutes). If BioNumerics® ask if you want to assign bands directly say yes and continue (the peaks will then be marked; note this is different from the actual allele assignments, which will follow later). Now you should see an additional window "Fingerprint curve processing”, where each quadruplex and peaks are seen in each lane.
9. You can switch between this window and the main window via the BioNumerics® icon in the taskbar. If you are new to BioNumerics® you may want to get an overview of the data you are handling: go back to the main window and select the entries you are working with and sort by "Selected Entries" in the top of the main window. Select also the five relevant filenames (_LIZ, _6FAM, _VIC, _NET and _PET) on the right-side panel called "Fingerprint files" and choose "Selected Fingerprint files".


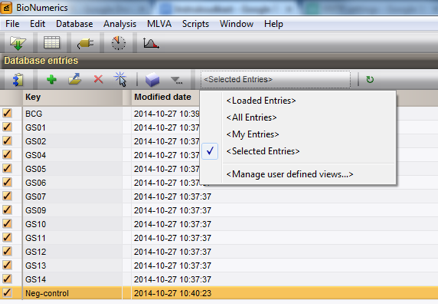


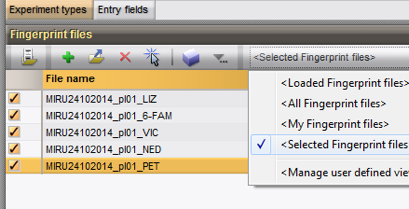


Reprinted from BioNumerics®, Applied Maths NV under a CC BY license, with permission from BioNumerics®, Applied Maths NV, original copyright 2016.

**Opening of previously imported data**

If you already started to analyse data, but have been away from the computer and must resume analysis, do this:

1. Open BioNumerics® via desktop icon.
2. Double-click on the relevant database.
3. Check the fingerprint files and mark just one of the relevant data files (best practice would be to mark and open for instance the LIZ file every time as you will then always get the colours in the same order when analysing the data). Click "Open the fingerprint data" (note, it may take a few minutes to load).


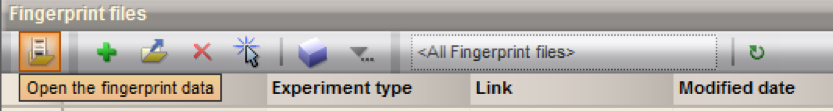


Reprinted from BioNumerics®, Applied Maths NV under a CC BY license, with permission from BioNumerics®, Applied Maths NV, original copyright 2016.

**Specifying standard sizes**

Go back to "Fingerprint curve processing". Sample ID and associated quadruplex are seen to the left and to the right you see the peaks. The orange are the standard sizes and the other colours represent a separate locus in a particular quadruplex for a given sample. At the bottom left you see "Channels", where you can choose to display only certain colours of the samples, for instance when you want to analyse your standard sizes only. You can zoom in and out on the cursor area by using Ctrl (vertical) or Shift (horizontal), while you scroll with the mouse. When setup correctly BioNumerics® defines standard sizes based on the settings the software has been fed with and is mostly spot on. But the standard sizes still have to be checked manually before proceeding with the allele calling. Therefore:

1. In "Channels" deselect all colours except the orange (use eye icons).
2. Already during the import, standard sizes from 80 to 1200 was assigned to all samples. For each lane, check that the standard sizes are correct by clicking on the triangles of peaks, which you will already know the size of (for example 80 and 240, 250, 260 and 840, 850, 860 and 1160, 1200) and check that the assignment is shown correct.


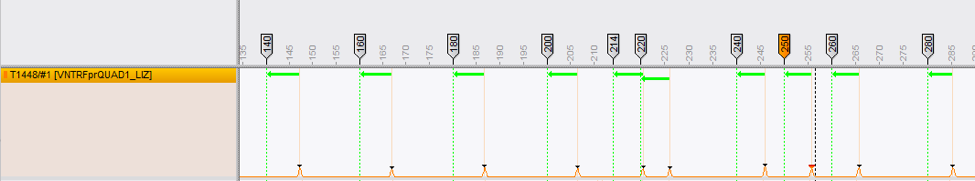


Reprinted from BioNumerics®, Applied Maths NV under a CC BY license, with permission from BioNumerics®, Applied Maths NV, original copyright 2016.

1. As a beginner, you should do spot checks of the standard sizes in each lane. By time, if everything just looks right, it may not be necessary to check all lanes systematically, since any errors should also be detected by systematically checking the allele assignments later in the procedure (where bins for each colour must fall in a regular pattern - if not, something is wrong with the standard sizes and they should be re-evaluated).
2. In rare cases the standard sizes are not correct and must be corrected. First delete all the assignments in the given lane under “Normalization” in the menu bar. The first known standard size is then selected from the right (i.e. from 1200 and down) and the actual size standard is selected in the series of numbers at the top. Then select "Normalization" in the menu bar and "Assign reference position". You have now manually assigned a standard size. Repeat with a couple more known size standard peaks and then select "Auto assign reference position" (current) ... "under" Normalization". Now, check if the standard assignments fit and remember to save in the menu bar. Note, if you have already begun the allele assignments you will need to close the "Fingerprint curve processing" window and reopen it via the relevant "Fingerprint File" in the main window for bins to change accordingly.

**Analysis of samples**

The allele calling is also done in "Fingerprint curve processing" - first by automatically calling them, then by doing a manual (sanity) check afterwards.

1. In the "Channels" deselect the orange colour and select all other colours.
2. Under "MLVA" in the menu select "Automatic assignment". When BioNumerics® asks which mapping to use, choose "Calibrated" as bins otherwise will not be positioned correctly. Let data load - it takes a moment.
   1. Note: if you select a peak (triangle) the size can be seen in the top left corner (“Band height"). In the upper right corner, you can see a list of samples (useful if you later want to check a particular sample).
   2. Note: In the left side in each lane, the highlighted colour indicates the bin positions in the given quadruplex (see screen dump below).
3. Now BioNumerics® has called alleles based on the data plus the information and settings we fed the software with. It's time to check the results:
   1. Note: each track is actually equivalent to the "raw data view” in Gene Mapper (other software for MIRU-VNTR analysis) with the relevant exception, that you can see bins for each of the 4 loci within a given quadruplex in a single frame, depending on the colour that is selected. In the upper left corner of the bins the associated allele number can be seen.
   2. Note: artefacts as pull-up peaks, stutter peaks, spurious peaks and shoulder peaks should be known - see Appendix I for details and examples. Double peak patterns should also be known - see Appendix II for a description and example.
   3. Again: zoom in and out by clicking Ctrl (vertical) or Shift (horizontal) while you scroll with the mouse. Note that you zoom according to where you placed your cursor.
   4. The triangle marks the peak - you can see the height of the peak in the top left corner.
   5. To delete a falsely called allele click "Ctrl-Shift-Delete", while you have the corresponding colour marked to the left of the lane. Alternatively, you can find the delete function via "MLVA" in the menu bar. It is tempting to delete an allele by simply selecting the peak and hitting "Delete”, but this is not the correct procedure, as the actual assignment is not deleted in this way.
   6. To call an allele mark the peak as well as the corresponding colour to the left and click "Ctrl-M" (again, this function can also be found via "MLVA" in the menu bar). If you see that a pull-up peak is falsely called and you also see the that the correct peak is not called, you can simply mark the true peak with “Ctrl-M” and then the falsely called peak will be deselected at the same time.
4. Start from first lane and work your way through all lanes. For each quadruplex, the true peak of each colour should be selected, i.e. normally four peaks in different colours marked per quadruplex. Be aware of the artefacts, especially pull-up peaks and stutter peaks, and make corrections accordingly.
5. To mark a double allele, select both peaks and then select "Manual assignment of selected peak (double)" under "MLVA" in the menu bar.
6. When you are done, click "Save" (at the top left) - very IMPORTANT. Go back to the main window.


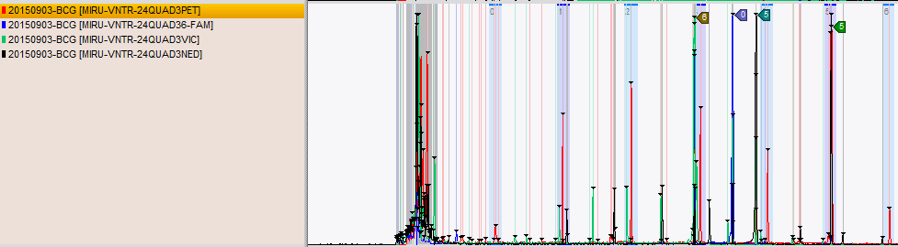


Reprinted from BioNumerics®, Applied Maths NV under a CC BY license, with permission from BioNumerics®, Applied Maths NV, original copyright 2016.

**Naming of genotypes**

Before naming the MIRU-VNTR types you may want to add a control step, where another trained person runs through the analysis. Continue with the naming hereafter:

1. In the main window, ensure that only the samples you are currently analysing is selected (with check marks).
2. Under "MLVA" in the menu bar, select "Perform MLVA typing...".
3. Say "OK" ("Perform typing") and let both MIRU_MtbC, MIRU_MtbC15 and MIRU_MtbC9 be chosen and say "OK" to name through the website miru-vntrplus.org subsequently ("Update").
4. You will now have the MIRU types if they exist, for example:
   1. “153-64” (profile is completely typed and already known by miru-vntrplus.org and thus named).
   2. “?-66” or “?-?” (profile is completely typed, but is not known by miru-vntrplus.org, i.e. it is a new genotype).
   3. “Incomplete profile” (profile missing alleles for one or more loci).
5. Changes: if data are re-evaluated and modified in the “Fingerprint curve processing” window remember to save. Then you can make a new naming of those samples in the main window (best practice is to select only the entries, that you want to rename). Select "MLVA" in the menu bar and "Perform MLVA typing...". The MIRU-profile should change accordingly.
6. New types: new genotypes are submitted to miru-vntrplus.org, so they can get a name. This is done directly in BioNumerics® by selecting the samples and then select "MLVA" and "Submit new MIRU-VNTR types" (you may want to add another step of control of the analysis before doing that).
7. Entries with double alleles: at present these cannot be submitted directly to miru-vntrplus.org. While they may be manipulated in the main window of BioNumerics®, it is probably easier to name them directly through miru-vntrplus.org, by first exporting data, manipulate the relevant locus in the exported spreadsheet to generate the two possible genotypes as reflected by the double allele, and then follow the genotype submission procedure on the website. The names may then be added manually in relevant columns in the main window of BioNumerics®.


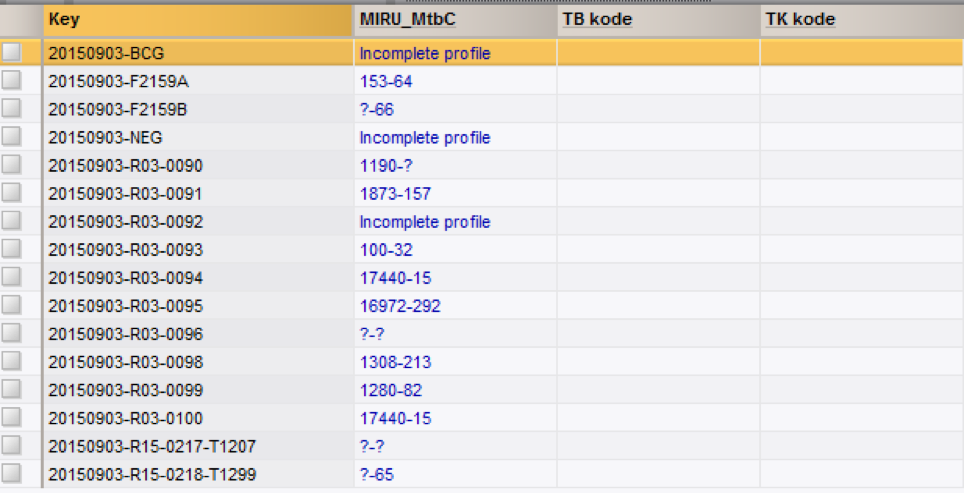


Reprinted from BioNumerics®, Applied Maths NV under a CC BY license, with permission from BioNumerics®, Applied Maths NV, original copyright 2016.

**Exporting data**

It may come handy to be able to export the data as a spreadsheet:

1. From the main window select "File" and then "Export" and then "Export fields and characters" and "Export".
2. Then select the following to export by holding down Ctrl and clicking with the mouse on "Key" + "MIRU-MtbC" + "MIRU-MtbC2" + "MIRU-VNTR_vals". Click "Next".
3. Select "Use mapping" and deselect "Active only" and click "Finish".

**Appendix I - Artefacts**

Pull-up peaks (observed frequently): are caused by the override of fluorescence from a very strong peak from one colour to another colour. In practice, it is observed that the true peak “pulls up” other colours, resulting in pull-up peaks that co-migrate with a true peak. To check for potential pull-up peaks the area around the peaks is enlarged (use zoom as described above). The true peak is assigned, while assignments from other peaks are removed (see how under “Analysis of samples”).


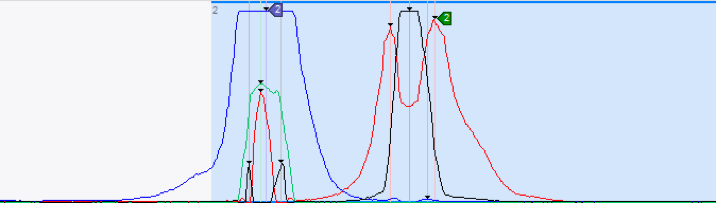


Reprinted from BioNumerics®, Applied Maths NV under a CC BY license, with permission from BioNumerics®, Applied Maths NV, original copyright 2016.

FIGURE 1: Pull-up peaks. On the left: the true blue peak pulls up green, red and black colour, which may be falsely allele assigned by BioNumerics®. On the right: the red peak pulls up the black peak. Only the true peaks should be assigned a repeat number (as shown in the figure).

Stutter peaks (observed frequently): are caused by slippage of the polymerase enzyme during PCR. The peaks can be easily identified, most often as a ladder of much lower peaks. The ladder corresponds to sizes of the PCR fragments that lack one or more repeats, or - less frequently - containing one or more additional repeats with respect to the true allele. In many cases, these stutter peaks will not be assigned by BioNumerics®. However, amplification of some loci with larger repeat numbers (e.g. locus 2163b and locus 4052), result in more intense stutter peaks that sometimes may exceed the height of the true peak and follow a "(half-)bell shape" distribution. In this case, a stutter peak may be falsely assigned instead of the true peak. This should be corrected: the true allele is the peak, which has the highest repeat number at the end of "(half-)bell shape”. After this peak, a sharp drop in the heights of following stutter peaks is usually seen.


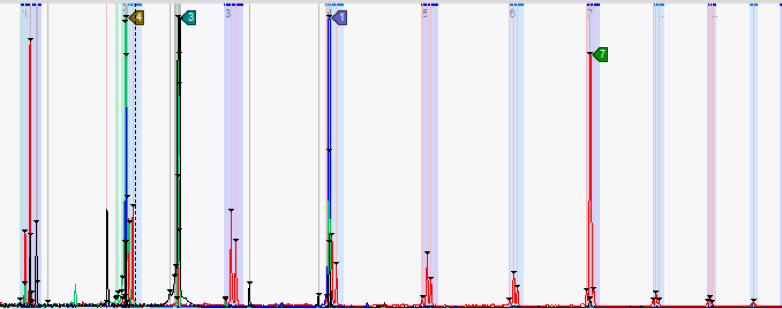


Reprinted from BioNumerics®, Applied Maths NV under a CC BY license, with permission from BioNumerics®, Applied Maths NV, original copyright 2016.

FIGURE 2: Stutter peaks. There are red peaks with decreasing intensity. The true peak is the end of "half-bell shape" (Bin 7) and not the peak of bin 1 even though this peak is actually higher.

Spurious peaks (observed very rarely): may be seen as thin peaks of multiple colours outside a bin. Can be caused by a bubble formation during migration of the fragments in electrophoresis. The peaks should not be assigned. But be aware if it is actually a double peak rather than a spurious peak (and also if the standard sizes are correct and thus have not caused a shift in the bins).


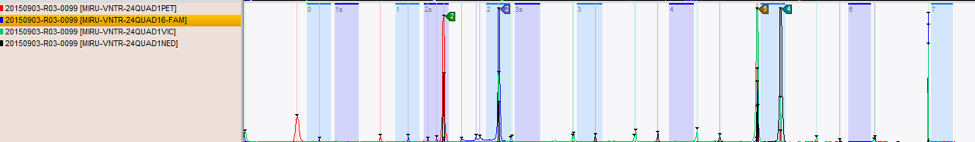


Reprinted from BioNumerics®, Applied Maths NV under a CC BY license, with permission from BioNumerics®, Applied Maths NV, original copyright 2016.

FIGURE 3A: Spurious peaks close to bin 7 for the blue colour.


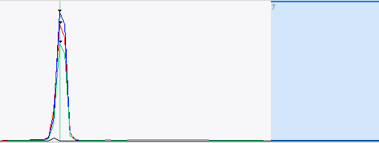


Reprinted from BioNumerics®, Applied Maths NV under a CC BY license, with permission from BioNumerics®, Applied Maths NV, original copyright 2016.

FIGURE 3B: Detail from Figure 3A, Spurious peaks.

Shoulder peaks (observed frequently, but less pronounced and rarely interfere with the analysis) may be seen as associated additional peaks to a major peak - often with pull-up peaks in other colours. They are caused by higher volumes of colour labelled fragments. Should not be assigned.


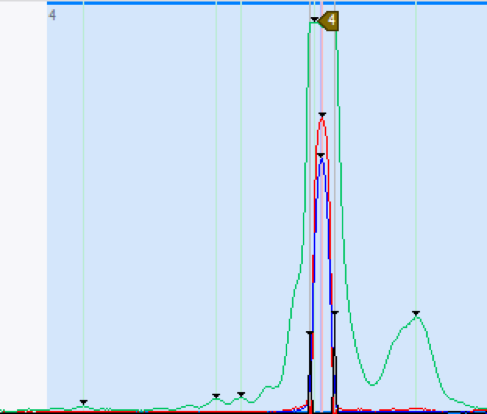


Reprinted from BioNumerics®, Applied Maths NV under a CC BY license, with permission from BioNumerics®, Applied Maths NV, original copyright 2016.

FIGURE 4: Shoulder peak to the right of the green peak, but still attached to the main peak (however, shoulder peaks are not necessarily attached to the main peak). Also, notice the pull-up peaks associated with the main peak.

**Appendix II - Double alleles**

Double peaks (observed occasionally) are not artefacts, but represents "ongoing evolution" reflecting two variants of the same clone, differing by a single locus. Double peaks appear as two true peaks, with heights clearly above that of (possibly present) stutter peaks and which cannot be explained by other artefacts (e.g. pull-up peaks).


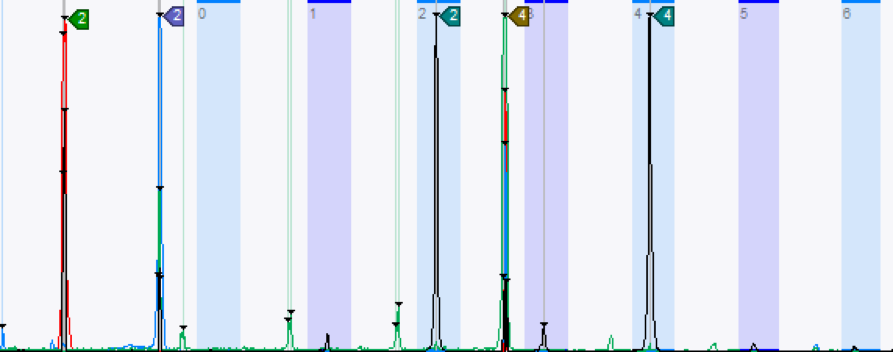


Reprinted from BioNumerics®, Applied Maths NV under a CC BY license, with permission from BioNumerics®, Applied Maths NV, original copyright 2016.

FIGURE 5: Double peaks in bin 2 and 4 for the black colour. Note that these peaks have heights that clearly surpass the heights of stutter peaks in the black colour present in bins 1, 3, 5 and 6. Double alleles represent "ongoing evolution", however always consider contamination, in particular if you observe what may apparently be double peaks in multiple samples from the same run.
